# The Current State and Usage of European Electronic Cross-border Health Services (eHDSI)

**DOI:** 10.1007/s10916-023-01920-9

**Published:** 2023-02-11

**Authors:** Jan Bruthans, Klára Jiráková

**Affiliations:** 1 Department of Biomedical Technology, Faculty of Biomedical Engineering, Czech Technical University in Prague, 272 01 Kladno, Czech Republic; 2 Department of Anesthesiology and Intensive Care, General Teaching Hospital, Prague, Czech Republic; 3 Information Technology Department, Regional Authority of Vysočina Region, Jihlava, Czech Republic

**Keywords:** eHealth, Electronic Prescription, Patient Summary, Interoperability

## Abstract

Introduction: European Union intends to enable cross-border health services through a program referred to as “MyHealth@EU”. The first main service is the dispensation of medicine by interlinking national electronic prescription systems. The second one is the Patient Summary, which enables providing the basic set of patients’ medical data. Methods: The contemporary technical documentation of the project was studied and selected published Key Performance Indicators of the project were analyzed. Where necessary, data were acquired directly from the European Commission. Results: Data from the start of the project (fourth quarter of 2019) until the second quarter of 2022 were analyzed. During this time both the overall number of EU countries with operational cross-border healthcare and their particular abilities in both services have risen. At present, there are eleven countries with capabilities in at least one of the services, of which nine have reported transactions. More countries are in the test phase now and will join the operational phase of the project shortly. Discussion and Conclusion: Nevertheless, the program is still used mostly for testing purposes. It seems that only electronic prescription and dispensation are commonly and widely used so far and only Estonian and Finnish patients usually get their medication dispensed abroad. The rest of the operational countries is still at present missing country pairs with a strong cross-border use case.

## Introduction

Although there is no general agreement about a single definition of eHealth [1], electronic health services are nowadays extensively used in most developed countries. With the rising mobility of mankind, the cross-border usage of health services comes to attention. This is most accented in the European Union, which nowadays forms the largest multinational entity consisting of 27 states with a total of nearly 447 million inhabitants. Some of the EU states (Denmark, Sweden, and the Netherlands) pioneered the first EPS nearly forty years ago [2] with a rapid spread over the last ten years. At present, most EU states have operational and widely-used EPS at a national level [3]. The same applies to EHR, which is at present functional at least in some EU states [4]. However, these national systems have developed independently and with no cross-border coordination. As a result, they differ significantly from each other. Although international standards (such as HL7) and coding systems (such as the International Classification of Diseases) exist, it seems not even two EU countries have interoperable national eHealth systems in place [5]. Moreover, using eHealth instead of paper-based documents might even complicate the delivery of care in another state (for example, a paper-based prescription would be accepted anywhere in the EU whereas the pharmacist in another state has no access to a foreign EPS and hence medication based on electronic prescription cannot be dispensed).

As the EU tries to unify many aspects of its national states (e.g., the Common Market of the EU, same citizens’ rights for every EU resident, etc.), there are also activities to connect national eHealth systems to enable cross-border healthcare in the EU. Hence a European Directive 2011/24/EU on patients’ rights in cross-border healthcare was adopted [6]. This is taken even further by a current proposal for a Regulation of the European Parliament and of the Council on the European Health Data Space, which, among others, presumes mandatory involvement of member states in the MyHealth@EU cross-border eHealth services.

Under the pilot European Patient Smart Open Services (epSOS) [7] running between 2008 and 2014 two cross-border services were selected – the dispensation of medication using EPS and Patient Summary (PS – a standardized limited set of patient health information to enable treatment by another physician). This pilot project transformed into an eHealth Digital Service Infrastructure (eHDSI) project financed under Connecting Europe Facility (CEF) [8] – a one billion € project running between 2014 and 2020. Now eHDSI falls under the EU4Health program [9] that will run until 2027. The cross-border services provided through eHDSI are referred to as “MyHealth@EU”.

eHDSI went operational in some European states already in 2019 and ever since is encompassing more states despite a recent delay due to the COVID-19 situation. Still, until now it has been mentioned in the scientific literature only marginally [10]. At present, there is no description of its current state nor of its current usage.

Therefore, this article aims to describe the current state of MyHealth@EU cross-border services running within the eHealth Digital Service Infrastructure, its present, and planned functions, and analyze selected statistics of its state and functions.

## Methods

eHDSI is documented on the periodically updated eHDSI confluence pages [11]. The three main domains are Operations (dealing with basic functions and overall usage), Sematic (dealing with interoperability of data), and Technical (dealing with technical aspects of the system, and the OpenNCP software). The entire website is freely accessible to everyone via EU Login (European Commission’s multifactorial user authentication services). Data about the system and its functions were gathered by analyzing the eHDSI confluence pages.

There is a large set of Key Performance Indicators (KPIs) of eHDSI, some of them still not monitored at present due to limited coverage of the services in the EU. All KPIs that are currently tracked are quarterly published on a public portal [12]. KPIs are divided into seven main groups, from which the first group KPI-1: Uptake is most reflective of the usage of eHDSI. The whole set of the KPI-1 group is set out in Table 1. We have excluded the KPI-1.6 analysis as it does not seem relevant to our study. Because our study is centered on eHDSI usage we have excluded KPI-1.9.1 to KPI-1.9.4., as they are more dependent on the context of individual states. KPI-1.10.1 seems rather speculative, so we have excluded it as well.

**Table 1:** Key Performance Indicators of eHDSI, group KPI-1: Uptake

KPI	KPI definition	Chosen for analysis
KPI-1.1	Number of Countries with Operational NCPeH	YES
KPI-1.2	Number of transactions between Countries	YES
KPI-1.3	Number of ePrescriptions exchanged	YES
KPI-1.4	Number of eDispensations exchanged	YES
KPI-1.5	Number of Patient Summaries exchanged	YES
KPI-1.6	Number of eDispensation Discard operations performed	NO
KPI-1.7	Number of Original Clinical Documents exchanged	YES
KPI-1.8.1	Number of Operational eP-A services	YES
KPI-1.8.2	Number of Operational eP-B services	YES
KPI-1.8.3	Number of Operational PS-A services	YES
KPI-1.8.4	Number of Operational PS-B services	YES
KPI-1.9.1	Pharmacies operational with eHDSI services	NO
KPI-1.9.2	Hospitals operational with eHDSI services	NO
KPI-1.9.3	Other Points of Care operational with eHDSI services	NO
KPI-1.9.4	A coverage percentage of the Points of Care operational with eHDSI services	NO
KPI-1.10.1	Number of citizens who are potentially able to benefit from eHDSI services	NO
KPI-1.10.2	Number of citizens excluding themselves from eHDSI services	YES
KPI-1.11	Number of citizens who have used the ePrescription service	YES
KPI-1.12	Number of citizens who have used the Patient Summary service	YES

The graphical way in which the KPIs are presented on the public portal is not ideally suited for further deeper analysis. Therefore, we have asked the European Commission, Directorate-General SANTE, Unit A4 to provide source KPI data, which we received as a list of all transactions in a set of spreadsheet documents.

## Results

Based on the above-mentioned EU directive 2011/24/EU only emergency, unplanned cross-border healthcare is considered. However, real-life practice is showing that it is not easily possible to differentiate between unplanned and planned use of cross-border healthcare, so the purpose of the use is not distinguished. The services differentiate between services from the point of view of “State A”, meaning a country in which the patient is domiciled and is usually treated, and “State B”, meaning a country in which the patient is treated at the moment or is trying to pick up medicine at the moment. So obviously, the typical use-case would be providing medical information from State A into State B (“pull” scenario). More complex scenarios, involving for example a “push” scenario from State B to State A, or even a third party State C, are currently not functional within eHDSI and are the subject of discussion for future use cases.

eHDSI, similarly to the former epSOS project, comprises from the beginning of two main services. The first one enables the dispensation of medicine using national EPS. This service is subdivided for technical reasons into ePrescription (eP, getting the information about the prescribed medicine from State A to State B) and into eDispensation (eD, getting the information of dispensed medicine from State B back to State A to rescind the prescription). Usually, this service is referred to as eP/eD. The second implemented service is the Patient Summary, which enables providing the basic set of patients’ medical data from State A to State B (where the patient is treated at that moment).

The crucial concept is achieving comprehensibility of the obtained data so that the healthcare professional in State B receives the information in his native tongue (as there are 24 official languages in the EU). eHDSI, therefore, has to provide for that via automatic mapping of the data in State A language to State B language. In every case, the document in original language (patient summary or ePrescription) is also available to the treating physician for possible use.

The newest addition to the eHDSI is the Original Clinical Documents (OrCD) exchange system. Under this service, original PDF versions of laboratory results, hospital discharge reports, medical images, and medical image reports will be exchanged. OrCD will remain in the original language and there will be no mapping of them.

As already stated above, the eHealth systems of individual European states are hardly compatible. eHDSI had been designed accordingly to overcome this situation. One National Contact Point for eHealth (NCPeH) must be provided in every member state. NCPeH has two interfaces – the national is the sole responsibility of the state whereas the international is standardized and developed jointly in the eHDSI project. So, the eHDSI project can be seen as an activity that encompasses close cooperation between a country NCPeH, and the European Commission, which is the owner of the eHDSI infrastructure.

To do so, a concept of Central Terminology Services was introduced. These services include, besides others, the Master Value Sets Catalogue (MVC) and Master Translation/Transcoding Catalogue (MTC), which are developed and maintained under the Sematic group. The MVC defines international coding systems used for the different data elements in the patient summary and eP/eD datasets (SNOMED CT, LOINC, ICD-10, etc.). The MVC serves as the basis for translations to each MS language and cross-reference between the selected code-systems, which results in the Master Translation/Transcoding Catalogue (MTC). Each member state is responsible for translating all the valuesets from the MVC and also performing any necessary mapping between international standards and standards used on the national level. During the document exchange transaction, medical data from State A are transcoded and translated to a commonly-agreed English format (“pivot document”), which is then sent to State B, where it is translated and mapped to State B language. Thus, it is ensured that data between two NCPeH are always in a coded and structured format. Member states are fully responsible for the document exchange process (translation and transcoding). In case a value set is not available in a coded format, it is provided to the treating healthcare professional at least in the original narrative text.

Another important issue is the authentication of patients and healthcare professionals. NCPeHs operate between themselves in the so-called “circle of trust”. This means that the authentication and authorization of a healthcare professional are always handled locally in State B. However, identification data of the patient have to be passed from State B to State A, where the decision is made whether the patient authentication is successful.EHR and EPS of individual European States differ also in the identification of the patient. Therefore, what type and range of patient identification need to be entered in State B is always defined by State A (as this is the country the patient is from). The information about type of information necessary for successful patient authentication is updated and shared between member states through configuration services.

The previous sentence explains just one of the reasons why eHDSI has to be treated not as an international system into an NCPeH of a particular country is integrated, but more like a point-to-point connection. The communication between member state NCPeHs is tested regularly throughout a complex testing framework, both in pre-production and operational environments. When a new NCPeH is introduced into operation, the communication with all other existing NCPeH has to be set up and tested one by one. Also, both scenarios (in one NCPeH works as State A and the other one when it works as State B) can be developed independently due to the different levels of maturity of the services in each state. This was predominantly seen in the beginnings of the system when it was for example possible to dispense medication from a particular State A in State B, but not the other way round.

**Fig 1:**
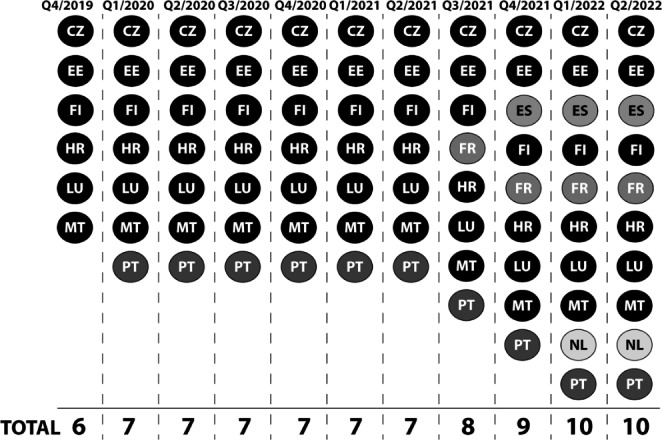
Countries with operational NCPeH, and its development in time Legend: CZ: Czech Republic, EE: Estonia, ES: Spain, FI: Finland, FR: France, HR: Croatia, LU: Luxembourg, MT: Malta, NL: the Netherlands, PT: Portugal

There were six countries with operational NCPeH (KPI-1.1) in the fourth quarter of 2019, the number has risen to ten at present – the evolution is set out in Fig. 1, different color is used to discriminate diverse quarters of introduction.

**Fig. 2:**
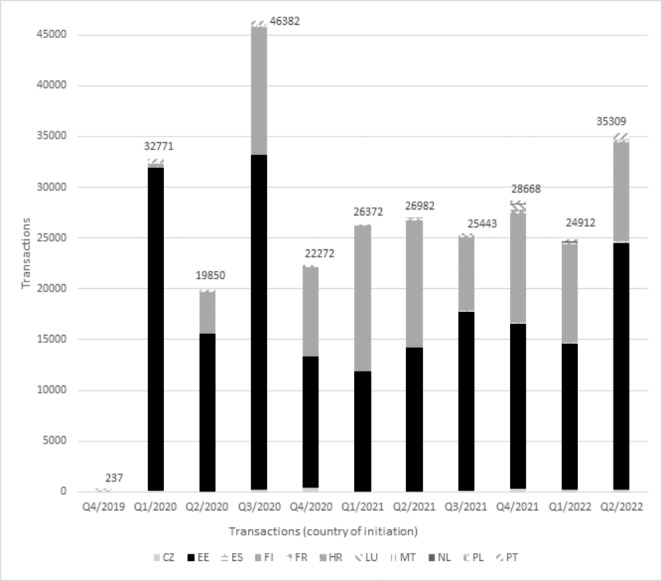
Transactions between countries Legend: CZ: Czech Republic, EE: Estonia, ES: Spain, FI: Finland, FR: France, HR: Croatia, LU: Luxembourg, MT: Malta, NL: the Netherlands, PL: Poland, PT: Portugal

The number of transactions (KPI-1.2) has risen from an initial 237 in Q4/2019 to 35.309 in Q2/2022. However, most transactions originate just from two countries – Estonia and Finland – as seen in Fig. 2.

Table 2 contains the number of ePrescriptions exchanged (e.g. request to gather data about prescribed medication) and the number of eDispensations exchanged (e.g. really dispensed medication). We have also added the eP/eD ratio in the table as this shows if the medication was truly dispensed. PIVOT format is seen as the primary in eP exchanges, so we regard the PDF format as complimentary, although theoretically PDF exchange can be also employed.

**Table 2:** ePrescription and eDispensation service

Country of initiation	Transaction type	Q1/2020	Q2/2020	Q3/2020	Q4/2020	Q1/2021	Q2/2021	Q3/2021	Q4/2021	Q1/2022	Q2/2022
Croatia	ePrescription – PDF format	19		20	6	4			6	6	5
	ePrescription – PIVOT format	15		18	2	2			34	26	11
	eDispensation – PIVOT format	10		6	1	1		2	3	1	4
	**eP/eD PIVOT to PIVOT ratio**	**0.67**		**0.33**	**0.50**	**0.50**			**0.09**	**0.04**	**0.36**
Estonia	ePrescription – PDF format	59	46	94	87	154	580	668	778	660	993
	ePrescription – PIVOT format	2673	3301	7423	2703	2632	2868	3371	3104	2840	5035
	eDispensation – PIVOT format	1683	1537	3721	1346	1108	1328	1652	1637	1516	2524
	**eP/eD PIVOT to PIVOT ratio**	**0.63**	**0.47**	**0.50**	**0.50**	**0.42**	**0.46**	**0.49**	**0.53**	**0.53**	**0.50**
Finland	ePrescription – PDF format	1	624	2016	1556	2628	2265	1385	2017	1783	1870
	ePrescription – PIVOT format	1	802	2320	1928	3316	3005	1638	2479	2213	2408
	eDispensation – PIVOT format	1	436	1545	1271	1953	1658	958	1449	1260	1279
	**eP/eD PIVOT to PIVOT ratio**	**1.00**	**0.54**	**0.67**	**0.66**	**0.59**	**0.55**	**0.58**	**0.58**	**0.57**	**0.53**
Poland	ePrescription – PDF format										34
	ePrescription – PIVOT format										68
	eDispensation – PIVOT format										10
	**eP/eD PIVOT to PIVOT ratio**										**0.15**
Portugal	ePrescription – PDF format	66	18	26		1			30	50	62
	ePrescription – PIVOT format		18	26		50			56	56	52
	eDispensation – PIVOT format	16		3					5	6	7
	**eP/eD PIVOT to PIVOT ratio**		**0**	**0.12**		**0**			**0.09**	**0.11**	**0.13**
Spain	ePrescription – PDF format										8
	ePrescription – PIVOT format										15
	eDispensation – PIVOT format										3
	**eP/eD PIVOT to PIVOT ratio**										**0.20**

The number of Patient Summaries exchanged (KPI-1.5) is set out in the same manner in Table 3. The OrCD exchange is still not introduced, so KPI-1.7 yielded no results.

**Table 3:** Patient Summary service

Country of initiation	Transaction format	Q4/2019	Q1/2020	Q2/2020	Q3/2020	Q4/2020	Q2/2021	Q3/2021	Q4/2021	Q1/2022	Q2/2022
Croatia	PDF		2	6	14	3			11		
	PIVOT		5	6	74	6		3	4		
Czech Republic	PDF		6		4	30	23	17	44	11	14
	PIVOT		38		60	88	37	43	10	26	36
Estonia	PDF								2	14	
	PIVOT								2	17	
France	PDF						14	34	111	19	13
	PIVOT						14	33	109	21	12
Luxembourg	PDF	21	10		6	8		2	35	11	3
	PIVOT	41	24		24	14		11	70	12	7
Malta	PDF	2	3		23	3			4		
	PIVOT	6	5		43				10		
Netherlands	PDF								6	3	
	PIVOT								12	6	
Portugal	PDF	2	12		13	8		7	5		4
	PIVOT	11	34		55	18		15	16	3	12
Spain	PDF							22	22	26	8
	PIVOT							11	4	16	18

**Fig. 3:**
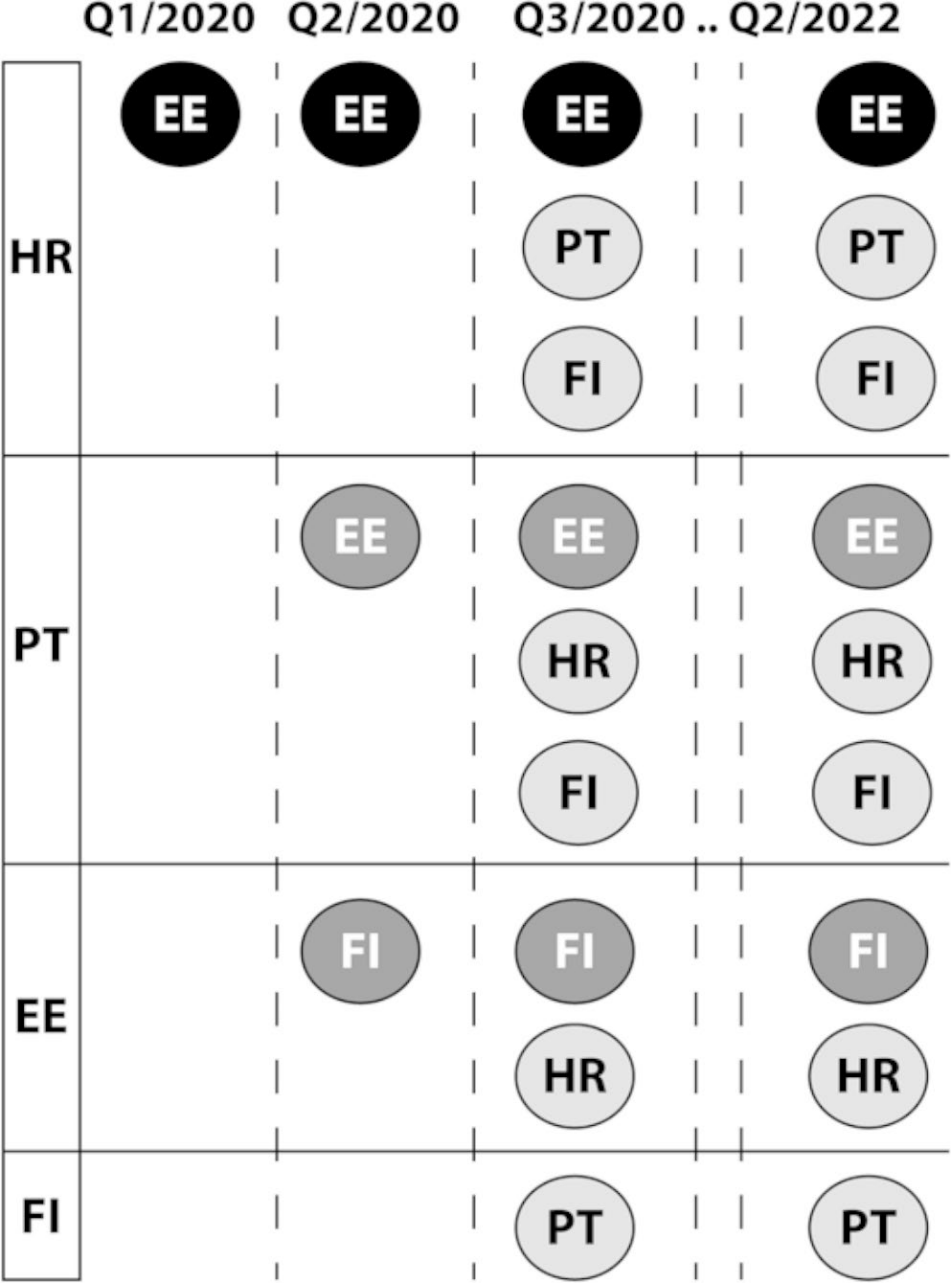
Countries with eP-A capability (left) and their corresponding eP-B capabilities and their development in time Legend: EE: Estonia, FI: Finland, HR: Croatia, PT: Portugal

As of Q3/2020, there were four countries with eP-A capability (KPI-1.8.1) and the same four countries with eP-B capability (KPI-1.8.2), although not all point-to-point abilities existed (e.g. Finland exhibited eP-B against Croatia, Portugal, and Estonia, whereas its eP-A capability existed against Portugal only) – the evolution and present state are set out in Fig. 3, again different colors are used to discriminate time of introduction.

**Fig. 4:**
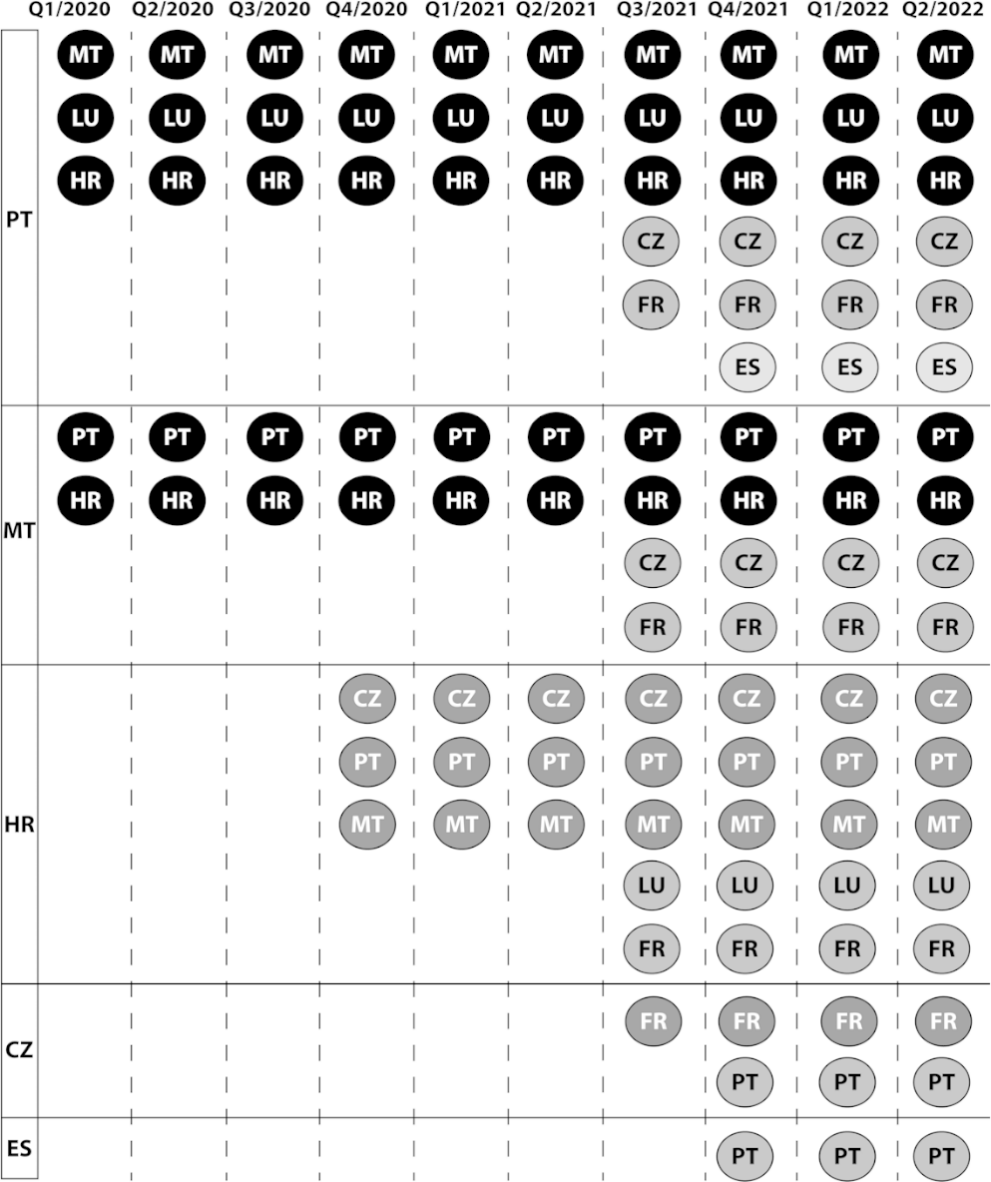
Countries with PS-A capability (left) and their corresponding PS-B capabilities and their development in time Legend: CZ: Czech Republic, ES: Spain, FR: France, HR: Croatia, LU: Luxembourg, MT: Malta, PT: Portugal

As of Q4/2021, there were five countries with eP-A capability (KPI-1.8.3) and two more countries with eP-B capability (KPI-1.8.4). Again, not all point-to-point capabilities exist – as seen in Fig. 4.

**Fig. 5:**
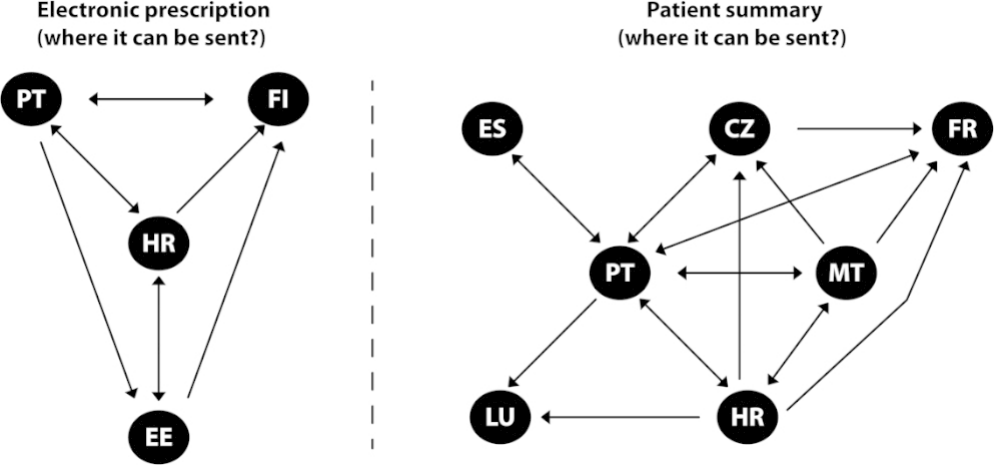
Chart of EU cross-border capabilities (arrow indicates the possibility to receive the data) Legend: CZ: Czech Republic, EE: Estonia, ES: Spain, FI: Finland, FR: France, HR: Croatia, LU: Luxembourg, MT: Malta, PT: Portugal

To clarify the point-to-point capabilities we have prepared an auxiliary Fig. 5 that depicts the real capabilities of every connected state – the arrowheads denote the State B capability.

KPI-1.10.2 to KPI-1.12 did not yield any usable results (the received data were scarce and rather haphazard), so we excluded them from our study.

## Discussion

Our study has some limits – firstly, not all transactions can be seen as “live” as they might be used just for test purposes. For that, a test attribute can be marked in a transaction. However, this feature is not used consistently so far. Therefore we have opted to include all transactions in our study. Secondly, there is no secondary source of the operational data of eHDSI, so we had no way to cross-check the available data.

The number of countries with operational NCPeHs has risen significantly in the studied period. The transactions between countries (KPI-1.2) mirror this – there are at least some for every NCPeH listed. However, the Netherlands still does not exhibit any live service (neither eP/eD nor PS as seen from KPI-1.8.1 to 1.8.4). Poland has begun its test transactions as well so we can expect this NCPeH soon.

The predominating transactions originating in Estonia and Finland are mirrored in the eP/eD, but there is much lower usage of PS until now. Therefore it seems only eP/eD service is commonly used so far and mostly only in Estonia and Finland. This can be explained by the frequent travels of their citizens between these two countries and thus a strong cross-border use case.

Also, there are no recorded PS transactions in Q1/2021 – the European Commission was not able to clarify this situation. The eP/eD ratio also shows that not every medication is dispensed as in many cases only the eP is used. This might show that the eP service is used for test purposes only (as opposed to eD when the real medication would be dispensed).

The number of operational eP-A services has risen from one in Q1/2020 (Croatia) to four in Q3/2020 and no other service was introduced ever since. Nevertheless, there are two more eP-A services in the test phase now (as seen from the eP/eD transactions). Similarly, the number of operational PS-A services has risen from two in Q1/2020 to five at present, with another four in the test phase now (as seen from PS transactions). The transactions also show us, that countries with State B capability only aim for getting the State A capability in the future as well.

No OrCD exchange is used, neither in testing nor as operational until now.

EU recommendations for interoperability have been thoroughly studied [13] with special attention to data protection [14]. Also, studies dealing with the introduction of interoperability in countries such as Finland and Estonia [15], and Italy [16] were also published. But we were unable to compare our findings with other literature, as the eHDSI operational data has not been analyzed so far.

## Conclusion

Nine EU countries have already shown some capability of cross-border health service – four actively exchange electronic prescription data (Croatia, Estonia, Finland, Portugal) and eight can exchange patient summary data (Croatia, Czech Republic, Finland, France, Malta, Portugal, Spain). We expect that the Netherlands (PS service), Poland (eP/eD service), and Spain (eP/eD service) will have this capability shortly as well.

However, it seems that only eP/eD service is commonly and widely used so far and only Estonian and Finnish patients usually get their medication dispensed abroad. The rest of the system (eP/eD in other countries and PS altogether) remains mostly in the testing phase, as the rest of the operational countries are missing country pairs with a strong cross-border use case, such as in the case of Estonia and Finland. The planned exchange of original clinical documents is not operational so far.

We observed a gradual spread of cross-border health service abilities, both in eP/eD and in PS. And although no new countries were introduced to the eP/eD service after the third quarter of 2020 and to the PS service after the fourth quarter of 2021, our data show that more countries will get this ability soon.

It may be concluded that although the cross-border MyHealth@EU services have a great potential to kick-start true patient-centered healthcare in the entire EU, where medical data with travel with/for EU patients, more widespread use will still take some years. Until now, the implementation of a national NCPeH and participation in cross-border services have been voluntary. With the coming of the EHDS Regulation, all EU countries will gradually become operational with cross-border services. It is expected that MyHealth@EU services will be frequently used between neighboring MS, as in the case of Finland and Estonia. It will be possible to provide reliable information to healthcare providers in their native language to ensure the continuity of healthcare. Besides patient summaries and ePrescriptions, the cross-border exchange of laboratory reports, discharge reports, images, and image reports is planned. To support patient empowerment, the access of patients to their translated medical data via MyHeatlh@EU is also envisaged in the short future.

## References

[1] Oh H, Rizo C, Enkin M, Jadad A, Powell J, Pagliari C. What Is eHealth (3): A Systematic Review of Published Definitions. J Med Internet Res 2005;7. 10.2196/jmir.7.1.e1. 10.2196/jmir.7.1.e1 PMC1550636 15829471

[2] Kierkegaard P (2013). E-Prescription across Europe. Health Technol.

[3] Bruthans J, Kofránek J, Vojtěch A (2021). Concept and practice of electronic prescription. Medsoft.

[4] Fragidis LL, Chatzoglou PD (2018). Implementation of a nationwide electronic health record (EHR): The international experience in 13 countries. Int J Health Care Qual Assur.

[5] Bruthans J (2020). The state of national electronic prescription systems in the EU in 2018 with special consideration given to interoperability issues. Int J Med Inf.

[6] Directive 2011/24/EU of the European Parliament and of the Council of 9 March 2011 on the application of patients’ rights in cross-border healthcare. vol. OJ L. 2011.

[7] Moharra M, Almazán C, Decool M, Nilsson A-L, Allegretti N, Seven M (2015). Implementation of a cross-border health service: physician and pharmacists’ opinions from the epSOS project: Table 1. Fam Pract.

[8] European Commission. Connecting Europe Facility. Innov Netw Exec Agency - Eur Comm 2015. https://ec.europa.eu/inea/en/connecting-europe-facility (accessed June 9, 2022).

[9] European Commission. EU4Health programme 2021–2027 – a vision for a healthier European Union 2021. https://ec.europa.eu/health/funding/eu4health-programme-2021-2027-vision-healthier-european-union_en (accessed June 9, 2022).

[10] Krastev E, Kovachev P, Tcharaktchiev D, Abanos S. Using QR Code for Uniform Representation of Content in Cross-Border Exchange of ePrescriptions in the EU. In: Mantas J, Stoicu-Tivadar L, Chronaki C, Hasman A, Weber P, Gallos P, et al., editors. Stud. Health Technol. Inform., IOS Press; 2021. 10.3233/SHTI210259. 10.3233/SHTI210259 34042663

[11] European Commission. My Health @ EU - eHealth Digital Service Infrastructure (eHDSI) Home 2022. https://webgate.ec.europa.eu/fpfis/wikis/display/EHDSI/My+Health+@+EU+-+eHealth+Digital+Service+Infrastructure+%28eHDSI%29+Home (accessed June 10, 2022).

[12] European Commission. eHDSI Monitoring Framework (KPIs) 2022. https://webgate.ec.europa.eu/santegis/eHDSI/ (accessed June 10, 2022).

[13] Bincoletto G (2020). Data protection issues in cross-border interoperability of Electronic Health Record systems within the European Union. Data Policy.

[14] Larrucea X, Moffie M, Asaf S, Santamaria I (2020). Towards a GDPR compliant way to secure European cross border Healthcare Industry 4.0. Comput Stand Interfaces.

[15] Palma FNS, Health ICDH (2022). Interoperability Challenges and Critical Success Factors in the Deployment of Cross-border Digital Medical Prescriptions in Finland and Estonia. 2022 IEEE Int. Conf. Digit.

[16] Nalin M, Baroni I, Faiella G, Romano M, Matrisciano F, Gelenbe E (2019). The European cross-border health data exchange roadmap: Case study in the Italian setting. J Biomed Inform.

